# Cost-Effective Network Reordering Using FPGA

**DOI:** 10.3390/s23020819

**Published:** 2023-01-10

**Authors:** Vinh Quoc Hoang, Yuhua Chen

**Affiliations:** Department of Electrical and Computer Engineering, University of Houston, Houston, TX 77204, USA

**Keywords:** FPGA, IoT, reordering, network

## Abstract

The advancement of complex Internet of Things (IoT) devices in recent years has deepened their dependency on network connectivity, demanding low latency and high throughput. At the same time, expanding operating conditions for these devices have brought challenges that limit the design constraints and accessibility for future hardware or software upgrades. These limitations can result in data loss because of out-of-order packets if the design specification cannot keep up with network demands. In addition, existing network reordering solutions become less applicable due to the drastic changes in the type of network endpoints, as IoT devices typically have less memory and are likely to be power-constrained. One approach to address this problem is reordering packets using reconfigurable hardware to ease computation in other functions. Field Programmable Gate Array (FPGA) devices are ideal candidates for hardware implementations at the network endpoints due to their high performance and flexibility. Moreover, previous research on packet reordering using FPGAs has serious design flaws that can lead to unnecessary packet dropping due to blocking in memory. This research proposes a scalable hardware-focused method for packet reordering that can overcome the flaws from previous work while maintaining minimal resource usage and low time complexity. The design utilizes a pipelined approach to perform sorting in parallel and completes the operation within two clock cycles. FPGA resources are optimized using a two-layer memory management system that consumes minimal on-chip memory and registers. Furthermore, the design is scalable to support multi-flow applications with shared memories in a single FPGA chip.

## 1. Introduction

The advancement in Internet of Things (IoT) devices has increased demands for new network technologies. Due to the broader usage of IoT devices, endpoints have to meet different bandwidths and latency requirements, depending on their applications [1]. At the same time, the operating environment for these devices has added new challenges to the design, including more constraints in power consumption, remote accessibility, limited memory, or processing capability [2,3]. These demands can lead to data loss in transmission due to network congestion and dropped packets. Since existing solutions to process packets are no longer economical in covering the unique traffic characteristics at each endpoint, finding a new approach for network processing tailored to IoT devices is essential.

IoT devices are collections of various sensors connected with actuators and processing systems to provide real-time data collection and data transfer [4]. They are commonly installed at the forefront of the data sampling environment, or the “edge”, connecting through a gateway to a more extensive network. Multiple device connections create high traffic for data transmission from the source back to the computing server, such as the cloud environment. As IoT devices start being used in broader applications, such as mobile networking devices, streaming services, or real-time artificial intelligence, they have to handle high bandwidth and achieve low latency to meet design specifications [5]. While devices can be designed to be more powerful, at the same time, the edge environment can constrain how much they can be expanded. In some cases, the environment can limit aspects of their physical designs, such as memory size and power consumption, or it can pose a challenge as to how to access the device for future upgrades in a harsh environment.

Cloud and edge computing are some of the many approaches to handle these constraints and reduce the network dependency of IoT devices. Edge computing devices, similar to IoT devices, are placed near the data-collecting hardware, with the addition of storage and real-time data processing capabilities before forwarding to the network layer [6]. The cloud computing model reads the sampled data and then processes them through the remote server [7]. Even though these methods ease some design burdens on the hardware, they rely on high-bandwidth networks, which is critical with the exponential development of machine learning applications in recent years, including on-demand image processing or computer vision [8,9]. The newer generation of devices with more stable connectivity, such as smart healthcare sensors, generates high volume of network traffic [10]. A network with limited bandwidth can experience congestion problems, resulting in data loss from out-of-order delivery, lossy links, or bit errors [11]. In addition, since these applications can carry critical content, there can be a damaging setback if the data traffic is not handled efficiently.

As a reconfigurable and custom design approach in the hardware domain, Field Programmable Gate Arrays (FPGAs) can be a suitable solution as the hardware network processing layer in edge and IoT devices. FPGAs have many uses in industrial applications because of custom digital logic design, which is popular in telecommunications, robotics, and automotive sectors, with practical applications from data centers to embedded devices [12]. They are readily available in many forms, including embedded soft processors, system-on-chip platforms, or development boards. The recent developments of FPGAs have resulted in higher performance and lower development cost [13], making integrating FPGAs into final IoT devices a possibility. In addition, the reconfigurable properties of FPGA have much potential for designing products with future upgrading needs.

As mentioned earlier, IoT and edge devices have covered a broader range of applications than ever. Therefore, it is crucial to ensure that real-time traffic is delivered with minimal loss or interruptions. One issue causing data loss for uncontrolled packet delivery, especially in low-latency applications such as data streaming, is out-of-order packet arrivals at network endpoints. Without proper processing, packets can be dropped, leading to unwanted delays and loss of critical data collection [14]. Packet reordering tasks are performed at the endpoint, traditionally servers and computers. However, the drastic change in IoT devices has made it more challenging to use standardized hardware to accommodate different traffic characteristics and design constraints. As the endpoint requirements have changed, packet reordering methods with a hardware focus should be revisited for modern IoT devices.

FPGAs have the potential to be applied to edge devices for packet reordering. Previous research has used FPGAs to reorder out-of-order packets for audiovisual streaming applications [15]. However, we identified significant flaws in this method. Their reordering technique optimizes resource usage, but it could create a situation preventing out-of-order packets from being sorted until the memory storing previous packets is freed. This design flaw can lead to unnecessary data loss and can only be avoided by doubling the memory size. The research and challenges of previous work are described in more detail in Section 2. Therefore, our research can overcome the flaws of earlier works and offer an efficient solution to sort out-of-order packets.

The motivation for this work is to address the packet reordering challenge for modern IoT development with unique design constraints. We propose a hardware method that can address the design flaws of previous solutions. At the same time, this method can be applied to different traffic characteristics of various devices, and it is flexible for future upgrades without the need to redesign.

The contributions of this paper are as follows:

- Providing a reconfigurable solution to packet reordering in hardware, which can be tailored for unique IoT applications.
- Allowing simultaneous insertion and release of packets, eliminating the design flaws in existing solutions.
- Inserting a packet in the correct order in two clock cycles with a runtime of O(1).
- Providing scalable support to multiple flows.

The rest of this paper is organized in the following order. Section 2 describes related research on FPGAs in network enhancement. Section 3 explains the proposed method and algorithms. Section 4 gives more details about the system architecture and functionality of the modules. Section 5 discusses the prototyping results and analyzes resource usage for different configurations with improvements to support multiple flows. Finally, Section 6 summarizes the method and future work, concluding the paper.

## 2. Related Work

This section summarizes the networking background for IoT devices, related works in FPGAs to improve connectivity, and some challenges arising from previous packet reordering research. Section 2.1 provides the overview of some research on applying FPGAs to improve IoT devices and network efficiency. Section 2.2 describes existing network reordering methods using FPGAs. Finally, Section 2.3 discusses the challenges and identifies design flaws of earlier works.

### 2.1. FPGA Research in IoT and Networking

Different factors, including network congestion, multiple flows, hardware or software bugs, insufficient bandwidth, or security issues, cause data loss. This can lead to severe consequences for real-time IoT applications such as autonomous driving, virtual reality, and health monitoring sensors. Mostacero-Agama analyzed the impact of latency on IoT devices and found that increasing the distance between the network providers and the receivers increased latency [16]. At the same time, higher latency led to higher jitters, impacting the response time and functionalities of IoT applications. Zhang found that deploying mobile edge computing facilities at the edge of the wireless network could effectively reduce the latency in IoT devices [17]. Their research used a machine learning algorithm to address the application-aware-edge-IoT problem. The machine learning model was designed to find the most effective policy for terminal devices–edge–nodes assignment to minimize the average latency between devices from unstructured input data. A paper by Hasan proposed information-centric networking over the common location-dependent and centralized network [18]. Their proposed architecture provided an efficient caching model for edge devices, resulting in faster content retrieval. The research emphasized the importance of having a robust design at the network endpoint for low-latency applications, avoiding data loss and optimizing performance.

In recent years, the uses for FPGAs have been transitioning from prototypes or small productions into final product integration. With the need for device updates over time, FPGAs offer a reconfigurable advantage compared with Application-Specific Integrated Circuits (ASICs). ASICs have higher performance, consume less power, and their design can be more optimized for a specific application. However, if a device needs modification, the hardware must go through manufacturing and reassembly, costing more time for deployment [19,20]. Recent FPGA technologies have been improved in terms of the fabrication process and enhanced development tools, closing the performance gap between the FPGA and the ASIC. Modern FPGAs have better energy efficiency, smaller real estate, and easier access to reconfiguration [21]. A recent survey of FPGA applications in IoT devices showed that FPGAs could benefit IoT devices by increasing network throughput, lowering latency, and improving network security [22].

The research by Yang took on the challenge of handling low-latency IoT applications using FPGAs [23]. They addressed the challenges of the current cloud computing model, which faces problems with latency and bandwidth due to the physical distance between the server and devices. They proposed a conceptual model for the FPGA, which could be used as an edge device for an intelligent vehicle-mounted system. The concept could provide real-time traffic and roadside information through a low-latency network environment. Research by Brasilino also implemented a hardware module to accelerate the application protocol for resource-constrained IoT devices [24]. The design can be synthesized as a system-on-chip FPGA, speeding up CPU performance by 308%.

To improve the flexibility of IoT hardware, Lee’s group developed a metamorphic IoT platform consisting of a cluster of connected edge computing devices that could be reconfigured on demand through a cloud network [3]. The edge device took advantage of both the ASIC and the FPGA in its architecture, where the ASIC was used as a processor, while the FPGA was designed as a reprogrammable hardware accelerator. The FPGA received the hardware configurations through the cloud server, allowing the flexibility of adjusting to edge-specific applications within the same hardware. In a different approach, other research from Aziz’s group proposed a solution to remotely reconfigure the FPGAs using a different architecture [25]. In their design, the FPGAs were used as part of the wireless sensor networks controlling the IoT environment. The new configuration was sent through the wireless Zigbee network in the form of a bitstream file to the flash memory. Their experiment resulted in lower data transmission overhead and power consumption.

Outside the scope of IoT, researchers have considered using FPGAs as replacements for CPUs in networking hardware. For example, Nakanishi used FPGAs to build a network-embedded system for TCP/IP without the need for a CPU [26]. They tested sending video signal packets from the camera to a display with two FPGAs, one for transmission and another for reception. Other research by Janković designed a system architecture on the FPGA for a space router in a satellite that could support a high bit rate of up to 100 Gbps [27]. This scenario showed an extreme operating condition in space where it was impossible to change the hardware mid-operation, and FPGAs could be an ideal platform for high throughput and flexibility. Their research used the high-end Xilinx FPGA, the Virtex Ultrascale+, to prototype the space router architecture consisting of parallel modules, yielding high throughput. These experiments have shown that FPGAs have the potential to be used as the sole network hardware itself, capable of handling high bandwidth demands.

### 2.2. Related Work in Packet Reordering

Network connectivity for IoT devices can be provided through different interfaces, for example, WiFi, 5G cellular, Bluetooth, or Ethernet. Even though more IoT-specified protocols have been developed, traditional network protocols such as Transmission Control Protocol (TCP) and User Datagram Protocol (UDP) are still applicable for many IoT applications. TCP is the preferred reliable protocol when slower network speed is not an issue. However, TCP can delay communication for applications that require low latency due to its three-way handshaking, which slows down the packet transmission and results in more power consumption [28]. Furthermore, with the parallel transfer of network payloads and random bit errors in wireless connections, TCP packets can arrive out of order, leading to data loss. UDP is more suitable for low-latency applications than TCP due to its faster speed for streaming, voice-over IP, or audiovisual multicasting services. The main disadvantage of UDP is that it does not offer flow control and tends to be unreliable. As a result, the transmission rate could surpass the receiver’s capacity, prompting packet drops. Researchers have explored alternative solutions for packet reordering using FPGAs to address the out-of-order packet problems in a new design environment.

One work focused on reordering was carried out by Zhou [29]. They proposed a method in the FPGA that worked for multiple TCP connections. Their algorithm stored two sets of TCP packet sequence numbers and data in two First In First Out (FIFO) memories. One of the modules verified whether the packet was in order by checking the ACK command as part of the TCP protocol. Then, the controller sent command signals to rearrange the sequence. The packet sequence number was sent to a pipeline and compared with other packets sequentially until it settled on a correct placement. Once packets were in-order, the controller sent a release command to the corresponding data from the memory to the application layer.

Another related work in solving the out-of-order problem is Beneš’s work on packet reordering [15]. Their research offered an approach to rearranging out-of-order packets in low-latency real-time transport audiovisual streaming applications. Their reordering scheme utilized the metadata of the payload instead of the full content to sort the packets, saving memory resources. First, an input module extracted metadata from the packets and stored them in DRAM. The metadata included the sequence number, the packet length, and the memory address in DRAM. The extracted sequence was sent to the reordering unit for sorting and stored in the reordering memory, where the address was determined by the lower bits of the sequence number. Before sending them to the output buffers, the reordering unit handled the flow of out-of-order packets using a “backpressure” mechanism to control the input and output logic for each packet. The backpressure mechanism used occupancy registers to read or write the metadata within the reordering memory. An output logic could only release the data if the correct in-sequence counter matched the sequence, validating that it was in order. Similarly, if the memory slot was already occupied, the input logic could not write new data until the output logic released it. Using this principle, packets sent to output buffers were in sequence. In the last stage, the output buffer used the metadata information of the in-order packet to retrieve the original payload from the memory. However, we identified a design flaw in this method, which we discuss in the following subsection.

### 2.3. Challenges

Existing solutions to packet reordering from the previous section offered innovative techniques in the hardware domain. However, there are some drawbacks to these works. Although Zhou’s method scales well with multiple TCP connections, its dependency on the ACK information to determine the in-order status makes it inflexible to other protocols. In addition, Zhou compared the sequence value in series within a set of predefined registers and comparators; this can increase the computing time required to place them in the correct location.

Beneš’s approach stores the metadata of the packets in the memory addresses indicated by the lower bits of the sequence numbers and releases packets when the memory slot of the expected sequence is filled. However, the scheme has a major flaw as it does not consider the time needed to retrieve packets that are in order. As a result, it can potentially discard packets as the memory slots might be occupied by packets already in order. For example, suppose the system supports the sorting of *N* packets, and Packets 2 to N−1 arrive before Packet 1. In this case, memory slots 2 to *N* are occupied. When Packet 1 finally arrives, it is written into memory slot 1. Now, we have all *N* packets that are in order. At this point, the system should be capable of accepting Packets in the range of N+1 to 2N. Unfortunately, all memory slots are filled, causing any out-of-sequence arriving packet to be discarded. It takes one (average) packet time to output one in-order packet. Therefore, future arriving packets are only accepted into the system if they are already in order. This flaw can be used by adversaries to launch Denial of Service (DoS) attacks.

Our method improves upon the foundations of the relevant reordering research and avoids loopholes in existing approaches. The goal is to minimize resource usage, allowing the flexibility to scale the flow to fit different network demands and applications. Furthermore, the design parameters are independent of specific protocols. Finally, we aim to achieve the time complexity of O(1) by taking advantage of the parallel processing capability in FPGAs. Our packet reordering approach is simple to implement and can be targeted to a wide range of FPGA devices.

## 3. FPGA-Based Network Reordering

This section describes the reordering method with our hardware design approach. Section 3.1 introduces the proposed method. Next, we provide a formal description of the proposed method along with the design constraints in Section 3.2. The formal definition ensures that developers who seek to implement the method can reproduce accurate results.

### 3.1. Proposed Method

**Figure 1:**
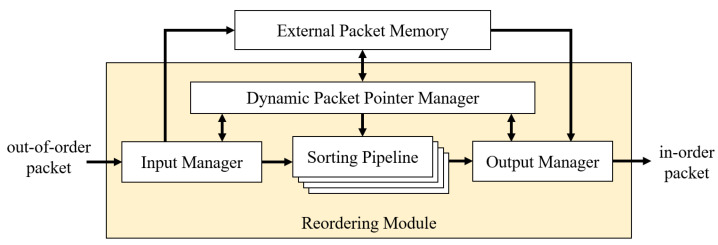
Top-level schematic of the proposed reordering method.

As mentioned earlier, the goal is to design a high-performance, cost-effective scheme to perform packet reordering. Our approach takes advantage of the hardware parallel shift registers to store data and reorder the sequence simultaneously with O(1) time complexity. We link these registers to create a pipeline where out-of-sequence packet references are stored and sorted in place. When a new packet arrives from the network, its sequence number is arranged in the pipeline in the correct ascending order within a fixed number of clock cycles. In our design, it is possible to achieve sorting and insertion in two clock cycles with the reordering module. Figure 1 shows the top-level system of this design.

The basic idea is explained as follows. Packets arrive from the network receiver and are fed into the reordering module. At the interface, we have an input manager that extracts the sequence from the packet while the packet is stored in the external memory. Only the sequence number and the reference pointer of the packet are passed to the sorting pipeline, which contains a series of shift registers. Each stage of the pipeline includes two fields: the sequence number and the packet pointer. The sorting pipeline has a property that the sequence numbers are always kept in ascending order. We use this property to identify the insertion position of the new packet in one cycle. After the packet is inserted into the correct place, the pipeline will remain in ascending order. Finally, the in-sequence packets are allowed to be retrieved by the output manager and forwarded to the network. During the sorting process, a dynamic packet pointer manager keeps track of the connections between the external memory addresses and the sequence numbers in the pipeline. This module translates the addresses into smaller-size reference pointers using an additional on-chip memory layer, indirectly linking the sequences to their contents in the external memory.

**Figure 2:**
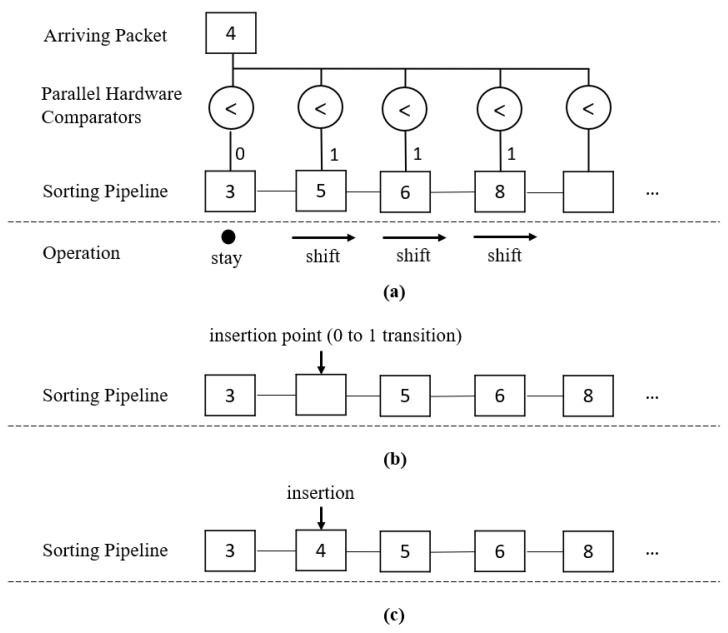
Example of the Sorting and Insertion: (**a**) Step 1: Identifying the insertion point when new packet arrives, (**b**) Step 2: Creating an empty spot in the pipeline after shifting, and (**c**) Step 3: Inserting the new packet into the pipeline.

Figure 2 shows an example of how it works in the sorting pipeline. For clarity, only the sequence number part of the pipeline is shown. In the example, the sorting pipeline has a few out-of-sequence packets waiting: Packets 3, 5, 6, and 8. Packets 2, 4, and 7 have not arrived. We assume that Packet 1 is in sequence and, thus, has been forwarded to the output. We then assume that Packet 4 arrives, as shown in Figure 2a. We must find the correct place to insert it and ensure that the pipeline is still in ascending order after insertion. In this case, the correct position for Packet 4 should be between 3 and 5. Registers containing Packets 5, 6, and 8 are moved toward the right side of the pipeline, while Packet 3 stays in place. As illustrated in Figure 2b, the pipeline now has an empty spot between 3 and 5, which is also the correct placement of Packet 4. This location is the insertion point, determined by the transition from 0 to 1 from the outputs of the hardware comparators. Inserting Packet 4 into this point gives us a new pipeline of Packets 3, 4, 5, 6, and 8 in ascending order. Figure 2c shows the final pipeline after insertion.

We only use the sequence number from the packet for the sorting pipeline without the original payload. The entire content is stored in the external memory, while the sequence number is extracted along with the memory address. However, using the full memory address as a pointer for the sequence can be costly. Therefore, we propose a scalable approach using a second memory layer in the FPGA, which is handled by the dynamic packet pointer manager. We use the addresses to this local memory as the reference pointer in our reordering module. Since the total number of out-of-order packets is limited, this drastically reduces the overall resource usage. The memory pointer arrangement is described in more detail in Section 4.3.

This proposed method can offer a scalable solution to the high-bandwidth network by using the reprogrammable capacity of the FPGA to adjust the size of the sorting pipeline. The pipeline size defines the maximum number of sequence numbers to be sorted; in other words, it is also the total number of available shift registers. Increasing the size allows more out-of-order sequences to be sorted while waiting for the in-sequence packet to arrive. The reordering module uses minimal resources to achieve a large pipeline size for applications requiring high network throughput.

It should be noted that the proposed method in this example shows a single flow. However, it can be scaled to multiple flows using multiple sorting pipelines connecting to a shared memory resource, as discussed in Section 4.4.

### 3.2. Formal Problem Definition and System Method

This section describes the problem definition and our formal method to address it. The problem definition is as follows: Let *U* be the sequence of packets with arriving time $t_{i}$, and $t_{1}<t_{2}<\ldots <t_{i}<\ldots <t_{n}$ for 0<i≤n, where the sequence number $U_{i}$ is greater than the sequence number $U_{j}$ of some packet with $t_{j}>t_{i}$. The objective is to output an in-order packet sequence regardless of the input sequence *U*. The method requires sorting the set of arriving out-of-ordered packets in ascending order within the pipeline.

The formal parameters and symbols used to describe the method can be found in Table 1. The sorting pipeline *P* containing *M* stages of shift registers is defined as $P=[P_{1},P_{2},\ldots ,P_{M}]$. Each stage $P_{i}$ stores a tuple ($S_{i}$, $B_{i}$), where $S_{i}$ is the sequence number, and $B_{i}$ is the corresponding packet reference pointer. The size of *M* is determined by the span, which is the maximum out-of-order packet distance. Since there are no duplicate sequence numbers, the values of $S_{i}$ are unique for 1≤i≤M. The invariant of the algorithm is that the sequence numbers are maintained in ascending order: $S_{i}<S_{i+1}$ for 1≤i≤M−1. The two components of the tuple in each stage are always shifted together. The incoming packet $U_{j}=\left(U_{s},U_{b}\right)$ carries the sequence number $U_{s}$ and reference pointer $U_{b}$. The sequence number $U_{s}$ of the incoming packet is used to determine the insertion point in the pipeline. The tuple $\left(U_{s},U_{b}\right)$ is inserted into the correct pipeline stage such that the sequence numbers remain in ascending order.

We first find a critical point *k*, which separates the pipeline stages into two sets: $P_{L}$ where $S_{i}<U_{s}$ for 1≤i≤k−1, and $P_{R}$ where $S_{i}>U_{s}$ for k≤i≤M−1. Because the span is *M*, there are at most M−1 packets waiting to be reordered when the incoming packet arrives. Therefore, the last stage $P_{M}$ is not occupied. This step takes one clock cycle in the FPGA hardware.

After determining the position *k*, the next step is to create an empty spot in the pipeline to insert the tuple $\left(U_{s},U_{b}\right)$. In the actual hardware, shifting the pipeline and inserting Packet $U_{j}$ are completed concurrently in one cycle. Here, we describe them in sequence for clarity. The tuples stored in the set $P_{R}$ are shifted to the right by one stage, making room for insertion at pipeline stage $P_{k}$. The tuple $\left(U_{s},U_{b}\right)$ of Packet $U_{j}$ is inserted into pipeline stage $P_{k}$. The shift registers in the pipelines have the property that they can be shifted in parallel. Depending on the value of *k* and the command to the sorting pipeline, we can shift the set either to the left or to the right.

There are three cases to operate the sorting pipeline: inserting a new packet, releasing a packet from the pipeline, or both. We describe the insertion, release, and concurrent insertion and release methods as Algorithms 1–3, respectively.
**Algorithm 1** Procedure to insert a new packet $U_{j}$.   Find *k* such that $S_{i}<U_{s}$ for 1≤i≤k−1, and $S_{i}>U_{s}$ for i≥k   for k<i≤M in parallel       $P_{i}\leftarrow P_{i-1}$; $P_{k}\leftarrow U_{j}$                ▹ Shift $\left[P_{k},P_{k+1},\ldots ,P_{M-1}\right]$ to the right; Insert $U_{j}$ in $P_{k}$

**Algorithm 2** Procedure to release a packet.
   if $C_{exp}==S_{1}$
       $O_{buf}\leftarrow S_{1}$
       for 1<i≤M in parallel
          $P_{i-1}\leftarrow P_{i}$; $P_{M}\leftarrow \left(INF,INF\right)$    ▹ Shift $P_{2}$ to $P_{M}$ to the left; Mark $S_{M}$ unoccupied
          $C_{exp}++$                                                             ▹ Increment expected in-sequence counter


**Algorithm 3** Procedure to insert a new packet $U_{j}$ and release a packet concurrently.
   Release $S_{1}$ to output and insert packet $U_{j}$ in pipeline
   if $C_{exp}==S_{1}$ and $U_{j}$ arrives
       Find *k* such that $S_{i}<U_{s}$ for 1≤i≤k−1, and $S_{i}>U_{s}$ for i≥k
       $O_{buf}\leftarrow S_{1}$
       for 1<i≤k−1 in parallel
          $P_{i-1}\leftarrow P_{i}$; $P_{k-1}\leftarrow U_{j}$                  ▹ Shift $P_{2}$ to $P_{k-1}$ to the left; Insert $U_{j}$ at $P_{k-1}$
          $C_{exp}++$                                                   ▹ Increment expected in-sequence counter


Algorithm 1 describes the inserting procedure for the new packet arrival. When the new sequence is not the expected in-sequence value, the procedure is executed to insert the tuple $\left(U_{s},U_{b}\right)$ in the correct order in the pipeline. The values stored in the set $P_{R}$ are shifted to the right by one stage.

Algorithm 2 describes the packet-releasing procedure, assuming no packet arrives. The algorithm keeps an expected in-sequence counter $C_{exp}$, which is the sequence number of the next in-sequence packet. This counter value is compared with $S_{1}$, which is the sequence number stored in pipeline stage 1. If the two values are equal, this means that the expected packet sequence has already arrived, and it should be sent to the output $O_{buf}$. The tuple stored in pipeline stage $P_{1}$ is sent to the output while the remaining pipeline stages shift to the left by one position.

Algorithms 1 and 2 describe the procedures for inserting an out-of-order packet and releasing an in-sequence packet separately. However, we want to achieve the highest throughput possible in high-speed networks. Algorithm 3 describes the procedure of handling the two cases concurrently. The intuition is described as follows. When a packet is released from the pipeline stage $S_{1}$, the rest of the pipeline should shift to the left to fill in the gap. If we want to insert the incoming packet *U* into the pipeline simultaneously, we only need to shift the pipeline stages to the left up to the critical point k−1, while keeping pipeline stages $P_{k}$ to $P_{M}$ unchanged. In this case, the pipeline stage $P_{k-1}$ is the insertion point for $U_{j}$. The procedure is described in Algorithm 3.

The key feature of the sorting pipeline is that it maintains the ascending order after the placement of $U_{j}$ with the shifting operations. We show the correctness of the algorithms.

Let $S=[S_{1},S_{2},\ldots ,S_{M-1}]$ be the sequence numbers stored in pipeline stages $P_{1}$ to $P_{M-1}$. Let *k* be the critical point, where $S_{i}<U_{s}$ for 1≤i≤k−1, and $S_{i}>U_{s}$ if any for i≥k.

**Theorem** **1.** *Inserting the value $U_{s}$ into the critical point k position in S keeps the sequence S in ascending order*.

**Proof.**  Base step: It is trivial to show that the condition is satisfied when *S* is empty. When the first element is inserted, it will be placed in the first location $S_{1}$. After the insertion, the single-element sequence *S* is also in ascending order.

Induction step: Suppose sequence $S=[S_{1},S_{2},\ldots ,S_{M-1}]$ is in ascending order before $U_{s}$ arrives. To insert the sequence number $U_{s}$, the critical point *k* breaks the sequence *S* into two sub-sequences: $S_{L}$, which contains sequence numbers up to $S_{k-1}$, and $S_{R}$, which contains sequence numbers from $S_{k}$ to $S_{M-1}$. Both $S_{L}$ and $S_{R}$ are in ascending order. Either $S_{L}$ or $S_{R}$ can be an empty sequence depending on the value of *k*. We analyze the following three cases.

- Case 1 (k=1): This means that all values in *S* are greater than $U_{s}$, or $S_{i}>U_{s}$ for 1≤i≤M−1. In this case, $S_{L}$ is empty and $S_{R}$ contains sequence $\left[S_{1},S_{2},\ldots ,S_{M-1}\right]$. The parallel shift operation shifts all elements in $S_{R}$ to the right by one position, increasing the indices of the values in $S_{R}$ by 1. Then, $U_{s}$ is inserted in position $S_{1}$. It is clear that the resulting $S=[U_{s},S_{1},\ldots ,S_{M-1}]$ is in ascending order.
- Case 2 (k=M): This means that $U_{s}$ is greater than all values in *S*, or $U_{s}>S_{i}$ for 1≤i≤M−1. In this case, $S_{L}$ contains sequence $\left[S_{1},S_{2},\ldots ,S_{M-1}\right]$, and $S_{R}$ is empty. The shift operation is essentially shifting zero element in $S_{R}$ to the right by one position. $U_{s}$ is inserted in position $S_{M}$. The new sequence contains $S_{L}$ appended with $U_{s}$, which is larger than any element in $S_{L}$ at the end. The new sequence $S=[S_{1},S_{2},\ldots ,S_{M-1},U_{s}]$ is in ascending order.
- Case 3 (1<k<M): In this case, the critical point *k* breaks sequence *S* into two non-empty sub-sequences $S_{L}$ and $S_{R}$, each of which is in ascending order. $U_{s}>S_{i}$ for all elements in $S_{L}$ and $U_{s}<S_{i}$ for all elements in $S_{R}$. The shifting operation increments the indices of all elements in $S_{R}$ by one: $\left[S_{k+1},S_{k+2},\ldots ,S_{M}\right]$. $S_{L}$ stays the same: $\left[S_{1},S_{2},\ldots ,S_{k-1}\right]$. $U_{s}$ is inserted in position $S_{k}$. Therefore, the resulting sequence $S=[S_{L},U_{s},S_{R}]$ is in ascending order.

We show that the pipeline remains in its ascending order regardless of the position of *k* in *S*. We conclude the proof of the theorem. □

## 4. Design

This section provides the details of how we implement the proposed method in hardware. Section 4.1 shows the system architecture and descriptions of the main modules. Next, we explain the sorting process in Section 4.2. Section 4.3 discusses optimizing memory resources by utilizing two layers of memory pointers. Finally, Section 4.4 describes how the design can be scaled to multi-flow applications.

### 4.1. System Architecture

**Figure 3:**
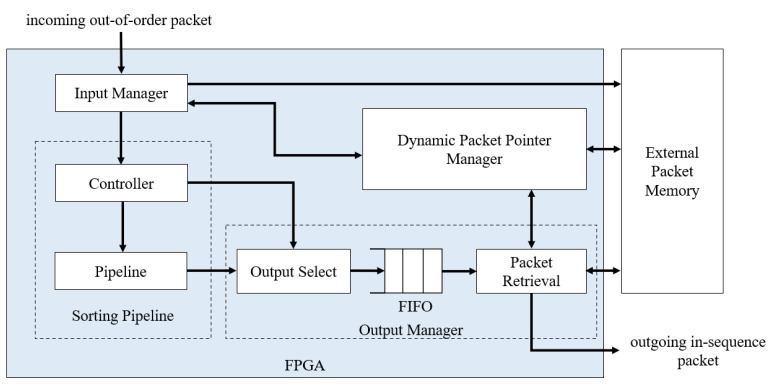
System architecture.

The top-level system architecture for the reordering scheme is built from four main modules: the input manager, the sorting pipeline, the dynamic packet pointer manager, and the output manager. The design is written using Verilog Hardware Description Language (HDL), which can be synthesized into any FPGA device. The system architecture in Figure 3 shows the connections between each module. The input manager and output manager communicate with the dynamic packet pointer manager to write and read the packet content.

The input manager is the front-end module of the FPGA that interacts with new packets coming from the network. As mentioned in Section 3.1, the sorting algorithm only needs the sequence number and the reference packet pointer to operate. The input manager handles this task. First, the module extracts the sequence number. At the same time, the dynamic packet pointer manager maps an unused external memory address to the on-chip reference pointer. Then, the external memory and on-chip reference pointer addresses are sent to the input manager. The input manager captures the external address and writes the packet content to the memory. Finally, the input manager forwards the sequence number and the reference pointer to the sorting pipeline.

The sorting pipeline module handles the sorting operations. Two values stored in the pipeline are the sequence number and the reference pointer; both values move together during the sorting process. Section 4.2 describes how the pipeline is designed in more detail.

Before sending packets to the network layer, the output manager retrieves in-order sequences from the sorting pipeline. At this stage, the output manager communicates with the dynamic packet pointer manager to translate the reference pointer to its address in the external memory. Using this address, the output manager reads the packet content from the memory. However, accessing data from the memory can experience a delay of several clock cycles during the read operation. To compensate for this, we introduce a FIFO at the output manager to send in-order sequences into a queue to be released one by one as soon as the read operation is completed.

### 4.2. Sorting Pipeline

The main feature of the sorting pipeline module is the ability to process all registers in parallel, different from iterations through each element in sequential programming. This feature helps achieve minimal sorting time; the new packet can be inserted and sorted in two clock cycles. To accomplish this, we connect a series of comparator blocks to each pipeline stage and compare the new sequence value $U_{s}$ to $S_{i}$ in parallel. The outputs from the comparators serve as an indicator that allows the controller in the pipeline to perform two tasks: finding the insertion position and identifying which register to shift.

The insertion position, *k*, is the first location where $U_{s}>S_{i}$, and the location is defined when there is a 0 to 1 transition in the comparator results. For example, we design the comparator block to generate 0 if $U_{s}>S_{i}$, and 1 otherwise. The controller in the sorting pipeline checks if there is a transition from 0 to 1 and locates the position *k*. Depending on the designer’s choice, they can invert output signals from the comparators. In addition, the comparator results are also used to identify which set is to be shifted. When a shift-right command is enabled from the controller, registers receiving an output of 1 from the comparator are shifted to the right toward the end of the pipeline by one position.

**Figure 4:**
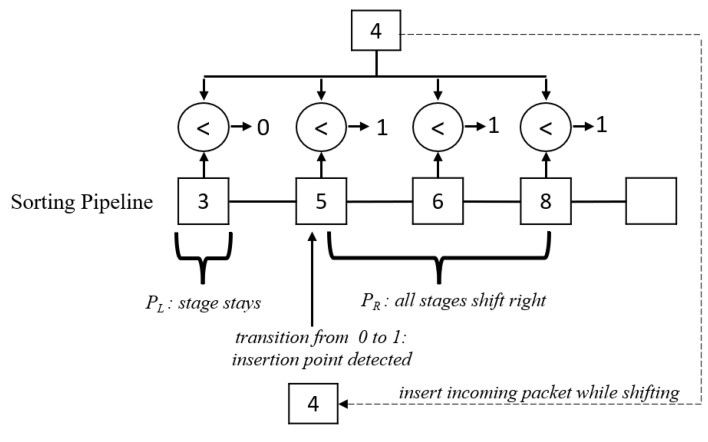
Implementation of comparator blocks to determine shifting operation.

Figure 4 illustrates an example of shifting and insertion using the comparator blocks. When the new Packet 4 arrives, it is compared to all packets in the pipeline. Since we have Packets 3, 5, 6, and 8 in the existing pipeline, a comparison with Packet 4 yields 0, 1, 1, and 1, respectively. The pipeline controller sees that the transition from 0 to 1 happens at position 2 in the pipeline, so it marks the critical point *k*. Then, all registers receiving 1 from the comparator outputs are shifted to the right simultaneously, resulting in an empty slot available for Packet 4. At the same time, Packet 4 is inserted into position *k*, taking advantage of the properties of hardware registers. The new pipeline contains 3, 4, 5, 6, and 8 after the operation.

The purpose of the controller within the sorting pipeline module is to send commands to perform the shifting and insertion operations in the pipeline. The commands enable shifting according to one of the three algorithms proposed in Section 3.2. To identify the appropriate shift operation, the controller keeps track of the in-order status of the sequences and oversees the flows between data coming from the input manager and the sorting pipeline.

We use a counter to store the expected in-sequence packet to check if a sequence is in-order. The counter starts from the first sequence and only increments when it equals the sequence value of the first stage register. Recall from Section 3 that the pipeline is always in ascending order. As a result, the minimum sequence is at the first position $S_{1}$. The controller decides whether to release the sequence based on the values of the counter and $S_{1}$. If they are equal, the controller sends a shift-left command to the pipeline, and $S_{1}$ is sent to the output manager. In the next cycle, the counter value is updated, and the checking process continues.

**Figure 5:**
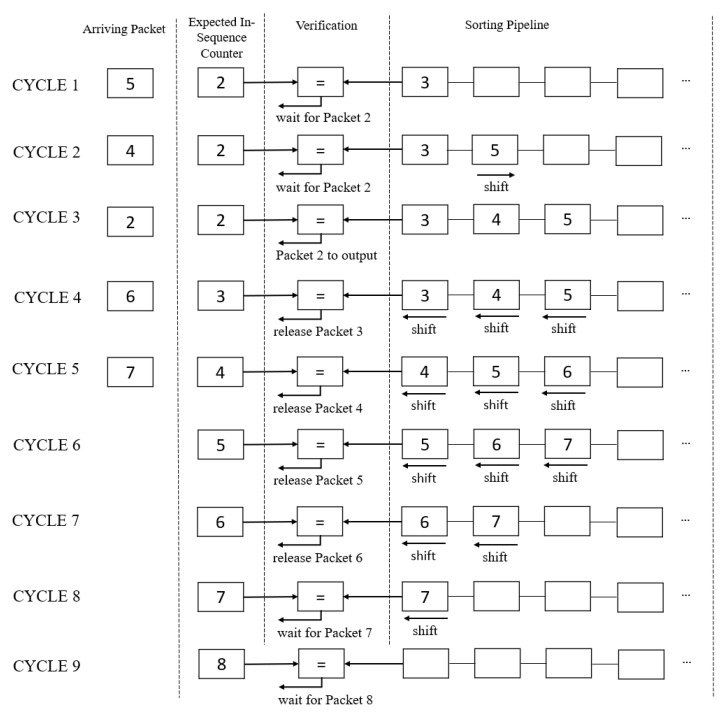
Example of using an in-order sequence to process the pipeline. Cycles 1–2 (Algorithm 1): Insert Packet 4 and 5 into the pipeline. Cycle 3 (Algorithm 2): Incoming packet matches the expected in-order sequence and is sent to output. Cycles 4–5 (Algorithm 3): Insert incoming packets and release the pipeline in parallel. Cycles 6–9 (Algorithm 2): Release the pipeline to output.

Figure 5 illustrates the counter implementation using either the release method, the insertion method, or both. In this example, Packet 4 arrives and is inserted into the pipeline after cycle 2, according to Algorithm 1. In cycle 3, Packet 2 is sent to the output according to Algorithm 2. In cycle 4, Packet 6 arrives, but the pipeline also needs to release Packet 3 to the output. This scenario triggers the release and insertion method to occur concurrently after cycle 4, following Algorithm 3. This is also the same for cycle 5. Cycles 6 to 9 show the example of Algorithm 2, where the expected in-sequence counter matches the pipeline, releasing them to output in each cycle until the pipeline is empty.

When an packet experiences a long delay, it may arrive much later in the flow or is not received at all in the case of a lost packet. To address this issue, there is a maximum waiting timer to trigger the counter increment to ensure the flow’s continuation.

In addition, the controller verifies the in-sequence status of the incoming packet from the input manager. This verification step lets the incoming packet bypass the sorting pipeline in two cases. The first case occurs when the incoming packet is already in order and the packet is sent to the output manager directly. The second case happens if the arrived sequence number is smaller than the expected in-sequence counter when the packet has a long delay. The delayed packet is stored in a separate memory location and sent to output afterwards. The decision of handling late packets is left to the end application.

### 4.3. Method for Memory Optimization

Following Beneš’s approach, we store the packet in the external memory and process the flow using only its metadata [15]. The benefits include reducing the data width in the design, allowing more resources in the FPGA to expand on other parameters, such as the size of the pipeline and the depth of FIFO. The TCP/IPv4 header can reach 100 bytes while the packet size itself in an Ethernet connection can be 1500 bytes [30]. Storing multiple packets can quickly overrun the on-chip memory block’s capacity in the FPGA. Therefore, storing the content in the external memory capable of handling large storage requirements, such as DRAM or SRAM, is beneficial. Even with this approach, address mapping to the external memory can become lengthy for the reference pointer to be stored in the sorting pipeline. For example, DRAM address mapping contains the three-tuple of bank, row, and column, and it can take up to a 64-bit DRAM address bus width [31,32].

**Figure 6:**
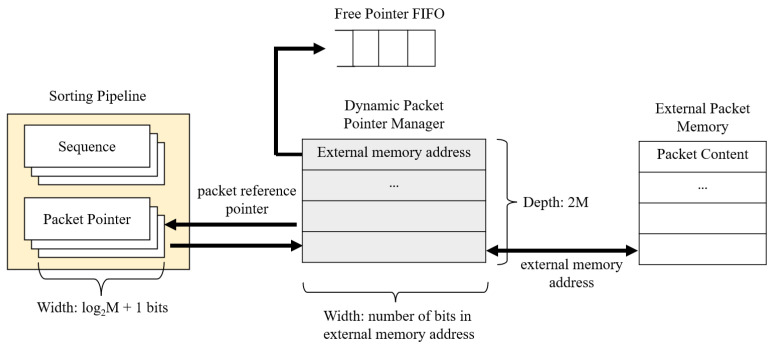
Memory optimization structure.

Storing long addresses in registers takes up resources. Hence, a proposed improvement capable of reducing data width for the pipeline’s address pointer is to use a second layer of FPGA’s internal memory pointing to the external address. The width of the internal memory is equal to the maximum number of bits in the external memory address, and its depth is 2M to accommodate in-order packets waiting for transmission. Therefore, the size of the packet reference pointer is $\log_{2}M+1$. For example, if *M* is 64, we only need to store 9 bits instead of 64 bits. This pointer scheme significantly reduces the size of each register in the FPGA compared with mapping the external address directly. The proposed approach avoids the flaw in Beneš’s approach. Figure 6 shows the memory management system.

To manage the internal address more efficiently, we create a FIFO to keep track of the free memory addresses dynamically. This method does not use linked lists; we can utilize any empty address as soon as one is freed up. Furthermore, since the dynamic packet pointer manager interfaces with the input manager, it can quickly provide the input manager with an available memory slot in the queue.

### 4.4. Multiple Flows Implementation

**Figure 7:**
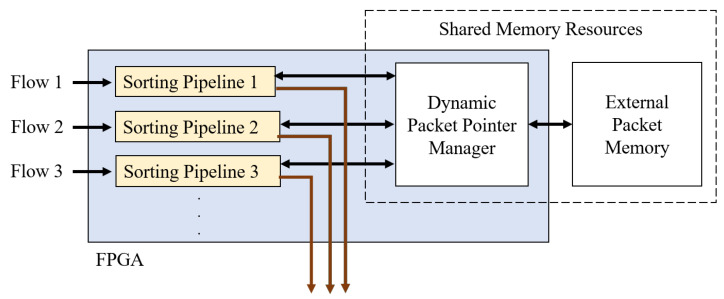
Multi-flow implementation using multiple pipelines.

The method described so far focuses mainly on a single flow between the sender and the receiver. However, the IoT consists of multiple devices constantly communicating. The multi-flow approach distributes the data from multiple sources, achieving higher throughput [33]. As a result, packets are separated into distinct channels, and there can be similar data loss challenges as in the single-flow case. A solution is to expand the methodology to a multi-flow solution by creating multiple instances of the sorting pipelines within one FPGA chip. This implementation allows each pipeline to reorder sequences in parallel within its data path. The required number of internal memory blocks increases with the number of flows, but both the on-chip memory and the external memory can be shared among all queues, as shown in Figure 7. Section 5.3 discusses the logic and memory utilization on a high-end FPGA chip using the multi-flow approach.

## 5. Prototyping and Experimental Results

This section provides the results from prototyping the reordering scheme on the FPGA hardware. First, Section 5.1 shows the detailed design implemented on the hardware. Then, Section 5.2 provides a snapshot of the signals captured from the circuit verification and details of FPGA hardware testing and verification. Finally, Section 5.3 analyzes the resource usage of this method and how varying the design parameter and expansion to multi-flow application affect the logic utilization.

### 5.1. Implementation Details

**Figure 8:**
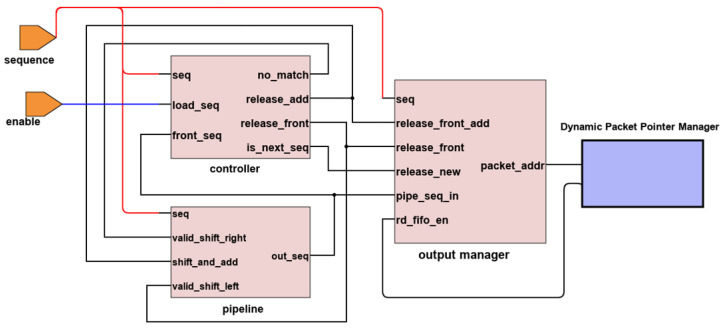
Verilog HDL module architecture for prototyping on FPGA.

This section describes how each module is implemented using the proposed method on the FPGA. The modules were written using Verilog HDL, utilizing Quartus Prime software to perform synthesis, transforming Verilog register-transfer level design to bit streams to be programmed onto the FPGA. Figure 8 shows the detailed design blocks as part of the sorting pipeline and the output manager.

FPGA devices contain dedicated blocks of on-chip memories, or block random access memories (BRAMs), with various sizes based on the FPGAs. We use the BRAMs as the internal memory resource storing the addresses of the external memory. BRAM of up to 32 Kbits each is becoming the standard, offering 32 bits of memory width and 1 Kbits of depth [34]. Beneš recommends the reordering range to be configured to 32, 64, or 128, based on their observation from the typical UDP reordering range [15]. A single BRAM is more than enough for our application without concatenation.

We do not show the input manager module and the external memory connections in order to simplify the explanation. The sequence number extracted from the input is fed into the pipeline controller for sorting. The counter is the internal register within the controller to keep track of the current in-order value. The controller compares the counter with the arriving sequence and the first register of the pipeline, as described in Section 4.2. The controller is designed using the finite state machine to send appropriate command signals to the pipeline or the output manager.

The shift registers in the pipeline are wired to its left and right neighbors to enable shifting. It is a standard digital design method to shift all data bits at once in one clock cycle. As mentioned earlier, we use comparator blocks as indicators for the pipeline. If the pipeline has *M* total number of registers, we generate *M* comparator instances to send comparison results to the registers.

The output manager uses a multiplexer to select the sequence released from the pipeline, or a by-passed value directly from the input manager, depending on the operation. The output from the multiplexer is fed into the FIFO queue. Only the BRAM address from the pipeline’s reference pointer matters at this level. The queue identifies the BRAM memory location, matches it with the external memory address, and retrieves the entire packet.

### 5.2. FPGA Prototyping

We implemented the proposed packet reordering scheme in the FPGA hardware using Verilog HDL and tested the design using the DE0-CV FPGA development board from Terasic.

We performed circuit verification with the ModelSim software to probe the digital signals to ensure that the outputs were as expected from a randomized set of inputs. We captured different states of the design and viewed the internal values at specific clock cycles in ModelSim.

**Figure 9:**
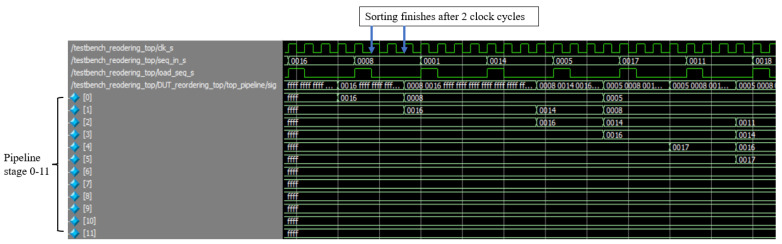
Circuit simulation and verification of reordering method using ModelSim software.

The signals shown in Figure 9 were captured from ModelSim, including the clock, the arriving sequence, the enable signal triggering a new sequence, and the first twelve stages of the sorting pipeline. All values shown were in hexadecimal format. The shift command sent a single-cycle high signal pulse, signaling that the sequence had arrived. After the enable signal, the finite state machine of the controller sent the shift-right operation in one clock cycle, and the sequence was inserted into the pipeline in the next clock cycle. The sequence value was successfully sorted and inserted in two clock cycles after the enable signal.

**Figure 10:**
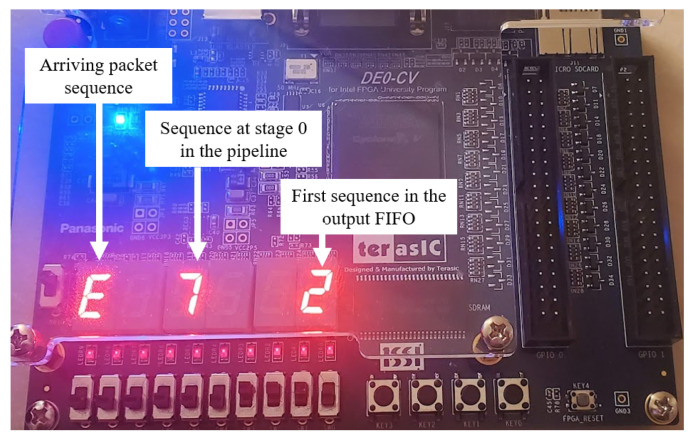
Testing the reordering method on FPGA hardware in manual mode.

We implemented the prototype on the Intel Cyclone V FPGA within the DE0-CV board for hardware testing and verification. The HDL modules were synthesized into the FPGA hardware using Quartus software. Timing analysis was performed to ensure that the design logic satisfied the hold and setup times with a 100 MHz clock. It should be noted that the hardware test was specifically set up to verify the packet reordering method. The packet sequence numbers were randomly generated and fed into the reordering module. Since packets were sorted every two clock cycles (50 million packets per second under the 100 MHz clock), we had two verification modes: automatic and manual modes. In the automatic mode, the output sequences were put into a FIFO, and a verification module was used to check the sequence for in-order delivery. In the manual mode, using the seven-segment displays on the FPGA kit, we could view the current input sequence and the values in the first stage of the pipeline. To verify the outputs, we used an enable switch button on the board to display the values stored in the output FIFO. Figure 10 shows the FPGA hardware used for testing the reordering method in the manual mode.

### 5.3. Resource Usage

The advantage of using the proposed method in FPGAs is its configurable features. We can reprogram the device to expand the pipeline size or the data width of the registers to fit different operational conditions. For instance, we can use a smaller pipeline size in a more stable network to save energy and resources. The opposite is also applicable if we expect many out-of-order packets with long delays. The modern FPGA architecture consists of adjustable logic modules (ALMs) as its main building block for reprogrammable complex logic functions. Increasing the data width, the number of BRAM blocks, the FIFO size, or the number of shift registers will take up more ALMs and on-chip memory bits. Choosing a suitable FPGA chip is crucial to strike a balance between cost and resources to meet the demand.

We compare the memory and ALMs utilization in FPGAs to analyze resource usage by altering various design parameters. The data are from synthesis results using the Quartus Prime Lite edition on the Cyclone V FPGA. For the baseline design, we use 16-bit sequence numbers with 32 shift registers to make up the sorting pipeline. The reference pointer will need 6 bits to map its BRAM address.

**Figure 11:**
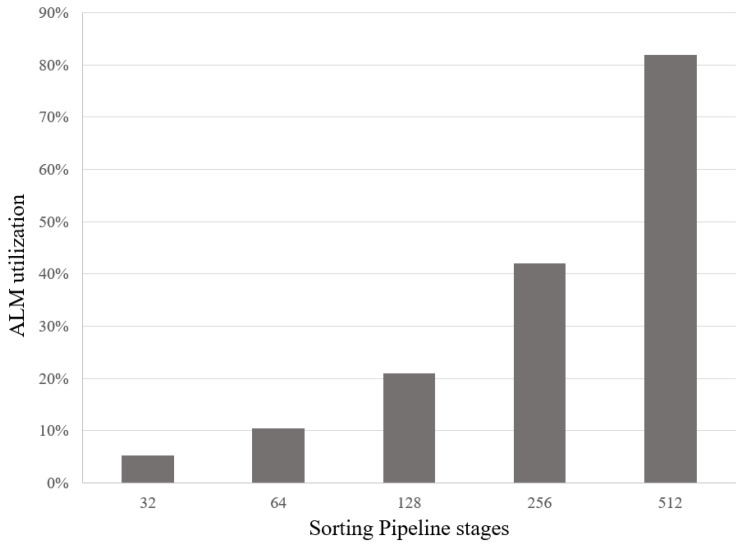
Analyze the ALMs resource usage in the Cyclone V FPGA when varying the total stages of the sorting pipeline from 32 to 512 stages with 16-bit packet sequence.

**Figure 12:**
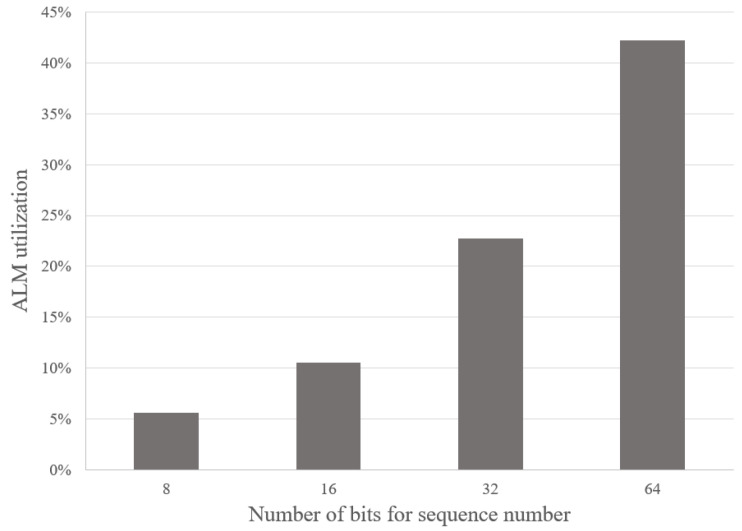
Analyzing the ALMs resource usage in the Cyclone V FPGA when varying the data width of the packet sequence from 8-bit to 64-bit for the pipeline with 32 stages.

Figure 11 shows the ALMs utilization in the FPGA when we increase the number of stages of the sorting pipeline. Increasing the total stages also increases the size of BRAM and FIFO needed. Other parameters, including the data width of the shift registers, are kept the same as the baseline defined above. With the Cyclone V FPGA chip, we can store up to 512 16-bit sequences, with 82% resource utilization. In Figure 12, we show how many ALMs are consumed if we vary the data width of the packet sequence in each pipeline stage. In this analysis, the total pipeline stages are kept the same as the baseline. The figure shows that we can expand the sequence data to 64-bit in the pipeline of 32 shift registers and only consume 42% of the resources in the Cyclone V FPGA.

Furthermore, we look into the resource utilization of the baseline design on a range of low-end to high-end FPGA devices, as shown in Table 2. Low-end FPGAs, such as the Intel Cyclone V commonly used for simple prototypes and educational development kits, consume about 11% of ALMs and still have sufficient memory space available. High-end FPGAs, such as the Intel Stratix and Arria series, introduce more ALMs, lookup tables, and logic elements. As a result, fitting the baseline design to the high-end device takes less than 1% of both memory and ALMs resources.

As discussed in Section 4.4, the design can be expanded to multi-flow applications. With more logic and memory resources for high-end devices combined with the small footprint of our method, we can see that high-end FPGAs such as the Arria 10 only consume 1% of ALMs and minimal on-chip memory blocks for a single flow. We use the Arria 10 for the experiment of fitting multiple sorting pipelines with the baseline specification of a 16-bit sequence number and a pipeline size of 64. Table 3 demonstrates the resource usage when the total number of sorting pipelines increases. The Arria 10 only consumes 13% of resources with up to 16 sorting pipeline modules, demonstrating that the design can sort beyond 16 flows in parallel and is scalable to multi-flow applications.

## 6. Conclusions

In this paper, we propose a scalable hardware-based approach to reorder packets for IoT devices using FPGAs. The proposed method can be implemented on reconfigurable hardware close to the endpoint for out-of-order packet processing, meeting the new design constraints of modern IoT and edge devices. It also overcomes the design flaws of the previous work on packet reordering. The reordering algorithm has a minimal time complexity of O(1), is scalable to multi-flow applications, and is simple to implement on FPGAs. Shifting and insertion operations in the sorting pipeline are completed in two clock cycles using the proposed method. When synthesized on a high-end FPGA, the design for a single flow uses less than 1% of both the ALMs and the block memory bits. In addition, we scale the design to fit multiple pipelines into one FPGA, and it takes less than 13% of ALMs when running 16 pipelines in parallel on an Intel Arria 10 FPGA. Furthermore, the design parameters, including the data width and the pipeline size, are adjustable to fit various operations and network demands. In the future, the reordering scheme can be synthesized to the endpoint of a network receiver. The fully integrated system can go through further field tests to gather more data on network traffic, monitor dropped packets, and accurately measure latency. Digital logic can be optimized further to ensure that the reordering scheme works with a higher frequency, meeting the demands of high-speed network applications.

In practical applications, this reordering method can benefit the vendors and customers in the industry. With the vast application of IoT in recent years, the network edges have also diverged from their traditional networking characteristics. It is costly to design hardware to cover all specifications or individual custom circuits for each device. Therefore, it is beneficial to move toward a reconfigurable hardware approach by taking advantage of the capability of future modifications for long-term usage. There is great potential to reduce overall costs in the device life cycle by using FPGAs as networking hardware.

## Figures and Tables

**Table 1:** Summary of parameters.

Symbol	Description
*P*	Sorting pipeline
*M*	Total number of stages of the sorting pipeline
$U_{s}$	Incoming packet sequence number
$U_{b}$	Incoming packet reference pointer
$U_{j}$	Incoming packet *j* containing the tuple $\left(U_{s},U_{b}\right)$
$S_{i}$	Sequence number in the pipeline stage *i*
$B_{i}$	Reference pointer in the pipeline stage *i*
*k*	Critical position point for insertion and shifting
$P_{M}$	Last stage in the sorting pipeline
$P_{L}$	Set of pipeline stages where $S_{i}<U_{s}$ for 1≤i≤k−1
$P_{R}$	Set of pipeline stages where $S_{i}>U_{s}$ for k≤i≤M−1
$C_{exp}$	Expected in-sequence counter
$O_{buf}$	Output buffer receiving in-order sequence from the pipeline

**Table 2:** Comparing ALMs and memory utilization for different types of FPGAs with a 32-bit packet sequence for the pipeline of 32 stages.

FPGA	ALMs Utilization	On-Chip Memory Bits Usage
Cyclone V	1941 (11%)	1024 (0.032%)
Stratix IV GX	2047 (2%)	1024 (0.007%)
Arria 10	1971 (1%)	1024 (0.002%)

**Table 3:** Comparing ALMs and memory utilization on Intel Arria 10 FPGA when increasing the total number of sorting pipelines in a single chip for multi-flow applications.

Number of Sorting Pipelines	ALMs Utilization	On-Chip Memory Usage
1	1%	0.002%
2	2%	0.005%
8	6%	0.019%
16	13%	0.038%

## Data Availability

Not applicable.

## Abbreviations

The following abbreviations are used in this manuscript:

BRAM	Block Random Access Memory
FPGA	Field-Programmable Gate Array
FIFO	First In First Out Buffer
HDL	Hardware Description Language
IoT	Internet of Things
TCP	Transmission Control Protocol
UDP	User Datagram Protocol

## References

[1] Xu Z., Liu W., Huang J., Yang C., Lu J., Tan H. (2020). Artificial Intelligence for Securing IoT Services in Edge Computing: A Survey. Secur. Commun. Netw..

[2] Liu J., Luo K., Zhou Z., Chen X. (2019). ERP: Edge Resource Pooling for Data Stream Mobile Computing. IEEE Internet Things J..

[3] Lee D., Moon H., Oh S., Park D. (2020). mIoT: Metamorphic IoT Platform for On-Demand Hardware Replacement in Large-Scaled IoT Applications. Sensors.

[4] Dawod A., Georgakopoulos D., Jayaraman P.P., Nirmalathas A. Advancements towards Global IoT Device Discovery and Integration. Proceedings of the 2019 IEEE International Congress on Internet of Things (ICIOT).

[5] Shafique K., Khawaja B.A., Sabir F., Qazi S., Mustaqim M. (2020). Internet of Things (IoT) for Next-Generation Smart Systems: A Review of Current Challenges, Future Trends and Prospects for Emerging 5G-IoT Scenarios. IEEE Access.

[6] Shi W., Pallis G., Xu Z. (2019). Edge Computing [Scanning the Issue]. Proc. IEEE.

[7] Botta A., de Donato W., Persico V., Pescapé A. (2016). Integration of cloud computing and Internet of Things: A survey. Future Gener. Comput. Syst..

[8] Long C., Cao Y., Jiang T., Zhang Q. (2018). Edge computing framework for cooperative video processing in multimedia IoT Systems. IEEE Trans. Multimed..

[9] Hussain T., Muhammad K., Ullah A., Ser J.D., Gandomi A.H., Sajjad M., Baik S.W., de Albuquerque V.H.C. (2021). Multiview summarization and activity recognition meet edge computing in IoT environments. IEEE Internet Things J..

[10] Aljubayri M., Peng T., Shikh-Bahaei M. (2021). Reduce delay of multipath TCP in IoT networks. Wirel. Netw..

[11] Kharat P., Kulkarni M. (2019). Congestion controlling schemes for high-speed data networks: A survey. J. High Speed Netw..

[12] Nasser Y., Lorandel J., Prévotet J.C., Hélard M. (2021). RTL to Transistor Level Power Modeling and Estimation Techniques for FPGA and ASIC: A Survey. IEEE Trans. Comput.-Aided Des. Integr. Circuits Syst..

[13] Machado R., Cabral J., Alves F.S. (2019). Recent Developments and Challenges in FPGA-Based Time-to-Digital Converters. IEEE Trans. Instrum. Meas..

[14] Xing Y., Xue K., Zhang Y., Han J., Li J., Liu J., Li R. (2021). A Low-Latency MPTCP Scheduler for Live Video Streaming in Mobile Networks. IEEE Trans. Wirel. Commun..

[15] Beneš T., Ubik S., Halák J. Packet reordering correction for low-latency network applications. Proceedings of the 2022 11th Mediterranean Conference on Embedded Computing (MECO).

[16] Mostacero-Agama L., Shiguihara P. Analysis of Internet Service Latency and its Impact on Internet of Things (IoT) Applications. Proceedings of the 2022 IEEE Engineering International Research Conference (EIRCON).

[17] Zhang L., Jabbari B. Machine Learning Driven Latency Optimization for Application-aware Edge Computing-based IoTs. Proceedings of the ICC 2022—IEEE International Conference on Communications.

[18] Hasan K., Jeong S.H. (2019). Efficient Caching for Data-Driven IoT Applications and Fast Content Delivery with Low Latency in ICN. Appl. Sci..

[19] Rodríguez-Andina J.J., Valdés-Peña M.D., Moure M.J. (2015). Advanced features and industrial applications of FPGAs—A review. IEEE Trans. Ind. Inform..

[20] Philip N.M., Sivamangai N.M. Review of FPGA-based accelerators of deep convolutional neural networks. Proceedings of the 2022 6th International Conference on Devices, Circuits and Systems (ICDCS).

[21] Alkhafaji F.S.M., Hasan W.Z.W., Isa M.M., Sulaiman N. (2018). Robotic controller: ASIC versus FPGA—A review. J. Comput. Theor. Nanosci..

[22] Magyari A., Chen Y. (2022). Review of state-of-the-art FPGA applications in IoT Networks. Sensors.

[23] Yang C. FPGA in IoT Edge Computing and Intelligence Transportation Applications. Proceedings of the 2021 IEEE International Conference on Robotics, Automation and Artificial Intelligence (RAAI).

[24] Brasilino L.R.B., Swany M. Low-Latency CoAP Processing in FPGA for the Internet of Things. Proceedings of the 2019 International Conference on Internet of Things (iThings) and IEEE Green Computing and Communications (GreenCom) and IEEE Cyber, Physical and Social Computing (CPSCom) and IEEE Smart Data (SmartData).

[25] Aziz S.M., Hoskin D.H., Pham D.M., Kamruzzaman J. (2022). Remote reconfiguration of FPGA-based wireless sensor nodes for flexible Internet of Things. Comput. Electr. Eng..

[26] Nakanishi A., Hatayama K., Onoduka T., Kimura T. An embedded system of TCP/IP communication by using FPGA. Proceedings of the 2015 IEEE 4th Global Conference on Consumer Electronics (GCCE).

[27] Janković S., Smiljanić A., Vesović M., Redžović H., Bežulj M., Radošević A., Moro S. (2020). High-capacity FPGA router for satellite backbone network. IEEE Trans. Aerosp. Electron. Syst..

[28] Chai L., Reine R. (2019). Performance of UDP-Lite for IoT network. IOP Conf. Ser. Mater. Sci. Eng..

[29] Zhou F., Hu Q. High-performance FPGA implementation of packet reordering for multiple TCP connections. Proceedings of the 2011 11th International Symposium on Communications & Information Technologies (ISCIT).

[30] Zhang B., Li Y., Liang Y. (2017). Impact of packet size on performance of TCP traffic with small router buffers. MATEC Web of Conferences.

[31] AMD Versal ACAP Programmable Network on Chip and Integrated Memory Controller LogiCORE IP Product Guide (PG313). https://docs.xilinx.com/r/en-US/pg313-network-on-chip.

[32] Wang M., Zhang Z., Cheng Y., Nepal S. DRAMDig: A knowledge-assisted tool to uncover DRAM address mapping. Proceedings of the 2020 57th ACM/IEEE Design Automation Conference (DAC).

[33] Thomas Y., Xylomenos G., Tsilopoulos C., Polyzos G.C. Multi-flow congestion control with network assistance. Proceedings of the 2016 IFIP Networking Conference (IFIP Networking) and Workshops.

[34] Pfeifer P., Pliva Z. On utilization of BRAM in FPGA for advanced measurements in mechatronics. Proceedings of the 2015 IEEE International Workshop of Electronics, Control, Measurement, Signals and their Application to Mechatronics (ECMSM).

